# PEGylated substrates of NSP4 protease: A tool to study protease specificity

**DOI:** 10.1038/srep22856

**Published:** 2016-03-09

**Authors:** Magdalena Wysocka, Natalia Gruba, Renata Grzywa, Artur Giełdoń, Remigiusz Bąchor, Krzysztof Brzozowski, Marcin Sieńczyk, Jenne Dieter, Zbigniew Szewczuk, Krzysztof Rolka, Adam Lesner

**Affiliations:** 1 Faculty of Chemistry, University of Gdansk, Gdansk Poland; 2 Faculty of Chemistry, Wroclaw Technical University, Wroclaw, Poland; 3 Comprehensive Pneumology Center, Institute of Lung Biology and Disease, Helmholtz Zentrum München, Munich, Germany; 4 Faculty of Chemistry, University of Wroclaw, Wroclaw, Poland

## Abstract

Herein we present the synthesis of a novel type of peptidomimetics composed of repeating diaminopropionic acid residues modified with structurally diverse heterobifunctional polyethylene glycol chains (abbreviated as DAPEG). Based on the developed compounds, a library of fluorogenic substrates was synthesized. Further library deconvolution towards human neutrophil serine protease 4 (NSP4) yielded highly sensitive and selective internally quenched peptidomimetic substrates. *In silico* analysis of the obtained peptidomimetics revealed the presence of an interaction network with distant subsites located on the enzyme surface.

Several useful methods to generate structurally diverse substrates to study the specificity of proteolytic enzymes currently exist^1^. In general, these substrates are short peptides containing a reporter chromogenic, fluorogenic or luminogenic group^2^. Specific peptide sequences are usually based on compounds naturally interacting with proteases and are further refined using enzyme crystal structure analysis as well as *in silico* studies^3^. Alternatively, high throughput screening of libraries composed of peptides or peptidomimetics yields optimized substrate recognition sequences^4^. This method has been routinely applied for the determination of protease specificity and the development of peptide-based protease probes^5^. A major limitation of this approach is the overlapping specificities of different proteases^6^. Closely related proteases with similar substrate recognition patterns are able to cleave similar peptide sequences at equivalent rates. In order to enhance the selectivity of such peptides, their original sequences are usually modified by replacing some of the proteinogenic amino acid residues with structural synthetic analogs^7^. Recently, Kasperkiewicz *et al*.^8^ described the development of a substrate library composed of non-proteinogenic amino acid residues. This library was validated against human neutrophil elastase and yielded a very potent fluorogenic substrate which was further converted into a specific activity-based probe.

Herein, we propose a novel and universal method to design and synthesize a library of peptide-like compounds composed of a series of diaminopropionic acid (Dap) residues substituted by heterobifunctional polyethylene glycol moieties. This library contains a Dap residue substituted on the amino group by a set of polyethylene glycol moieties at positions P2–P4, while the P1 position is fixed with a particular amino acid residue (in this case of human neutrophil serine protease 4 the P1 position is occupied by an Arg residue).

Our goal was to cover a broad range of chemical interactions between the enzyme and its substrate molecule. In each position, the chemical groups differed by side chain character specificity including amino, guanidino, carboxy, methoxy, hydroxy and carbobenzyloxy groups (basic, acidic, neutral, hydrophilic, hydrophobic) which was provided *via* the variety of functionalized PEG derivatives. Simultaneously, the effect of side chain length was investigated since three possibilities of length for each side chain were available: first, simple amino acid chain or its stable protected form; second, side chain extended by single ethylene unit form of all components; third, replication of chemical entities with two polyethylene unit extension. Importantly, the introduction of PEG residues made the prepared libraries highly soluble in water.

This approach guaranteed the selection of the optimal residue at each position (Fig. 1). Diversifying the library by inserting 18 different compounds at every X_2_–X_4_ position led to the generation of almost 6000 compounds covering a broad range of chemical space. The library was synthesized using manual solid-phase synthesis applying Fmoc chemistry and was screened using an iterative approach in solution. The release of ANB-NH_2_ quencher resulted in an increase of free ABZ-peptide fluorescence that allowed us to determine the optimal residue at each examined position. Incorporation of polyethylene glycol residue to the protein or peptide (selective PEGylation) resulted in improved water solubility and higher proteolytic stability of modified molecules; while increasing the molecular weight prolonged the half-life of conjugates^9^,^10^. Selective PEGylation has been successfully used for cross-linking of molecules^11^, synthesis of peptide-like polymers modified with different chemical entities^12^, generation of self-assembling nanostructures^13^, cell delivery of molecules, and many other applications (described in ref. 14).

Our target enzyme in this study was human neutrophil serine protease 4 (NSP4), recently discovered by Jenne *et al*.^15^,^16^. The amino acid sequence of NSP4 shares almost 39% identity with the sequences of human neutrophil elastase (HNE) and proteinase 3 (PR3). NSP4, together with HNE, cathepsin G (CatG), and PR3, is released into the pericellular environment as a result of neutrophil activation. Similar to other neutrophil serine proteases (NSPs), NSP4 is synthesized as an inactive precursor (preproNSP4) which, after the removal of signal peptide, is activated by cathepsin C. In contrast to other NSPs, NSP4 displays a strong preference towards an arginine residue at the P1 position of the substrate (according to Schechter-Berger nomenclature^17^), but its primary sequence forecasts a very different elastase-like active site with a preference for small aliphatic amino acids. This complication was recently unraveled by Lin *et al*. who solved the crystal structure of NSP4 complexed with d-Phe-l-Phe-l-Arg chloromethyl ketone^18^. Based on the provided data, the Arg residue is in a non-canonical “up” form stabilized by a network of hydrogen bonds involving the guanidinium group and Ser residue of the enzyme and hydrophobic shallow cleft that interacts with the aliphatic side chain of the Arg typical for elastase-like proteases. Due to this atypical substrate binding mode, NSP4 is able to recognize and process substrates containing arginine-like residues such as citrulline or methylated arginine.

## Results

**Figure 1:**
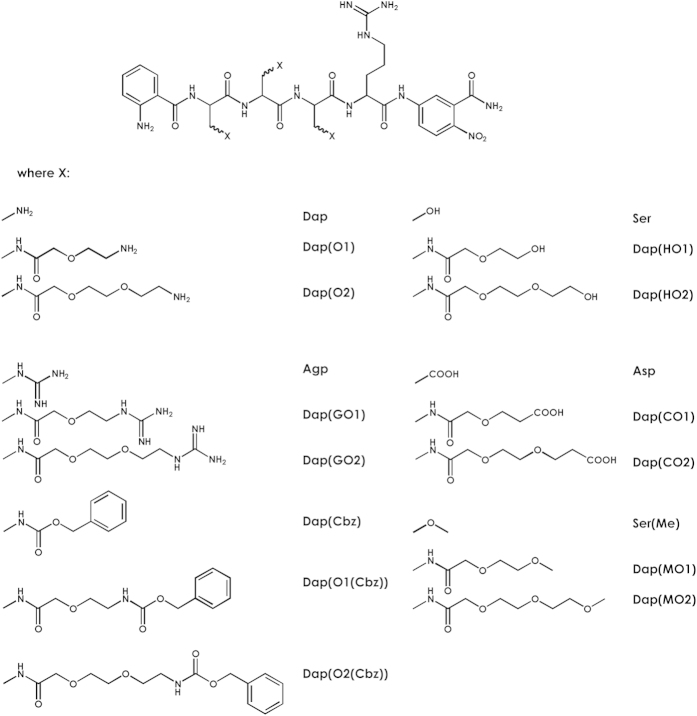
Overview of library and its components. 5-(*tert*-butyloxycarbonyl-amino)-3-oxapentanoic acid (further abbreviated as O1), 8-(*tert*-butyloxycarbonyl-amino)-3,6-dioxaoctanoic acid (O2), 5-[N-*tert*-butyloxycarbonyl-N′-(2,2,4,6,7-pentamethyldihydro benzofuran-5-sulfonyl)]amidino-3-oxapentanoic acid (GO1), 8-[N-*tert*-butyloxycarbonyl-N′-(2,2,4,6,7-pentamethyldihydrobenzofuran-5-sulfonyl)]amidino-3,6-dioxaoctanoic acid (GO2), 5-(benzyloxycarbonyl-amino)-3-oxa-pentanoic acid (CbzO1), 8-(benzyloxycarbonyl-amino)-3,6-dioxaoctanoic acid dicyclohexylamine (CbzO2), 2-(2-*tert*-butoxyethoxy) acetic acid (HO1), 2-(2-(2-*tert*-butoxyethoxy)ethoxy) acetic acid (HO2), 3,6-dioxaoctanedioic acid 1-*tert*-butyl ester (CO1), 3,6,9-trioxaundecandioic acid 1-*tert*-butyl ester (CO2), 5-methoxy-3-oxapentanoic acid (MO1), 8-methoxy-3,6-dioxaoctanoic acid (MO2) along with l-2,3-diaminopropionic acid Dap, Cbz-protected Dap, Ser, Ser methyl ether, l-2-amino-3-guanidino-propionic acid (Agp) and Asp.

**Figure 2:**
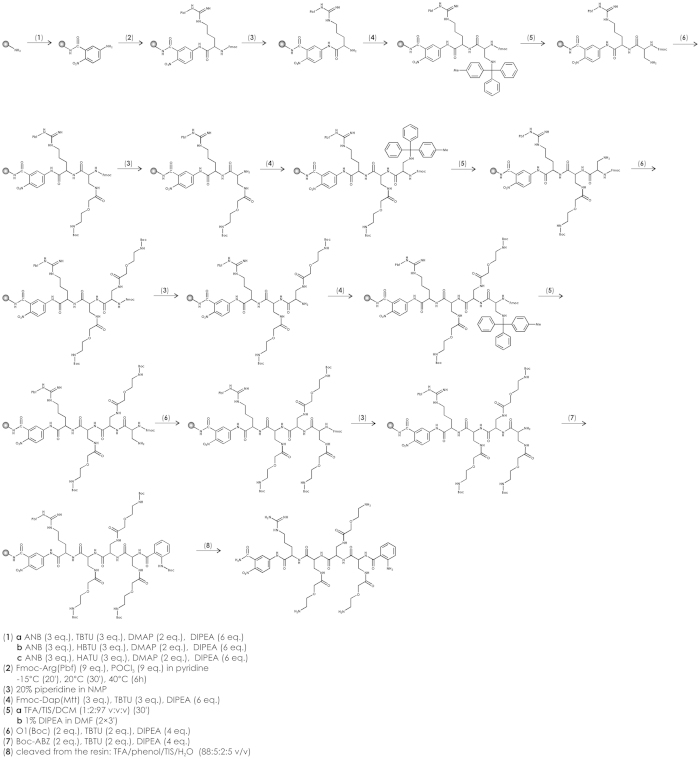
An example of a synthetic scheme for DAPEG-based peptidomimetic preparation.

In order to construct a novel type of internally quenched library, we used different monoprotected heterobifunctional PEG moieties to diversify a library based on a diaminopropionic acid scaffold (Fig. 1 presents the library formula and its chemical components). The library was synthesized using the mix and split method *via* Fmoc chemistry. The formation of Dap-PEG conjugates was performed on resin via a submonomeric method (one example of DAPEG-based molecule synthesis is presented in Fig. 2). Our goal was to investigate P2, P3, and P4 subsite preferences with a fixed P1 position occupied by an Arg residue. Each peptide in the library was flanked by a *C*-terminal amide of 5-amino-2-nitrobenozic acid (acceptor of fluorescence) and an *N*-terminal 2-amino benzoic acid (fluorescence donor) pair which allowed for simultaneous UV and fluorescence monitoring. Screening process was performed using an iterative mode of deconvolution in solution.

**Figure 3:**
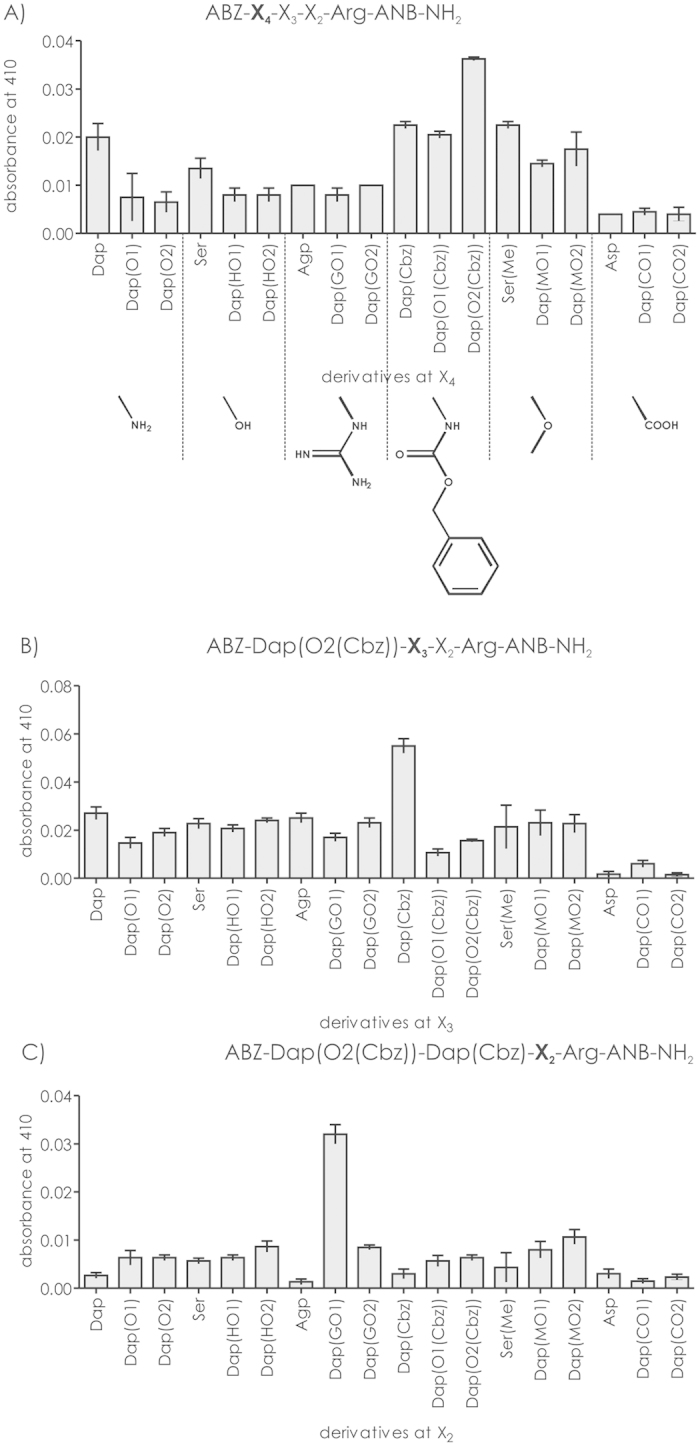
The deconvolution of the library (at average concentration 3.4 × 10^−5 ^M) with general formula ABZ-X_4_-X_3_-X_2_-Arg-ANB-NH_2_ against NSP4 (1.46 × 10^−8 ^M).

The 18 sublibraries with fixed X_4_ positions were incubated with NSP4. The highest hydrolysis rate of the Arg-ANB-NH_2_ bond was observed for diaminopropionic acid modified by carbobenzyloxy-protected amino polyethylene glycols Dap(O2(Cbz)) and Dap(O1(Cbz) (Fig. 3A) followed by the remaining amino acid residues - Dap and the Cbz-protected analog (Dap(Cbz)) and methyl ether of Ser. A relatively high rate of proteolysis was also observed of methyl ether derived PEG chains with supremacy of the longer Dap(MO2) chain - Insignificant output was observed for acidic residues despite side chain length. Considering the above findings, in next our step deconvolution of Dap(O2(Cbz)) was included yielding the ABZ-Dap(O2(Cbz))-X_3_-X_2_-Arg-ANB-NH_2_ library.

Next, 18 sublibraries with randomized X_3_ position were analyzed. In assayed position, the most intense proteolysis was observed for compounds with short side chain amino acid residues - Dap(Cbz), Dap and Agp (Fig. 3B). The highest rate of hydrolysis was observed for the residue with aromatic ring present on its side chain - Dap(Cbz). Other residues were poorly processed by NSP4 with 3–4 times lower hydrolysis rate. Importantly, the proteolytic efficacy of the best sublibrary was three times greater than the most potent residue found for the X_4_ position. Following analysis, Dap(Cbz) was included at position X_3_ resulting in an 18-member library with a general formula of ABZ-Dap(O2(Cbz))-Dap(Cbz)-X2-Arg-ANB-NH_2_.

Incubation of each of 18 peptides modified at position X_2_ with NSP4 indicated that Dap analogs substituted by PEG chains displayed the highest fluorescent intensity (Fig. 3C). Within this set, the PEG chain with guanidinium group was processed at the highest rate. Low proteolysis was observed for the set of short-chained amino acid - Dap, Dap(Cbz), Agp or Asp with the only exception being Ser and its methyl ether. The sequence ABZ-Dap(O2(Cbz))-Dap(Cbz)-Dap(GO1)-Arg-ANB-NH_2_ (further abbreviated as substrate 1) was selected as most susceptible for NSP4-mediated proteolysis.

A mass spectrometry quality check was performed at all stages of library deconvolution and all library components were identified within the HR MS spectra (Fig. S1). Similar analyses were performed for randomly selected sublibraries for each assayed position (X_4_, X_3_ and X_2_) identifying all signals corresponding to [MW+H]^+^ or its sodium/potassium adducts (Figs S9, S10 and S11).

The obtained substrate 1 was characterized in order to verify its chemical composition and sequence. The HPLC and MS analysis of substrate 1 indicated a purity >98% and a molecular weight equal to 1271.34. Formation of a well-defined y-ion series was observed following MS/MS fragmentation (see Figs S2 and S3), and these, together with the ions originating from Cbz loss/cleavage fully validated the structure of substrate 1. The structure of substrate 1 was further validated using the 2D ^1^HNMR method (details are provided in Supplementary Material, Figs S4, S5 and S6).

**Figure 4:**
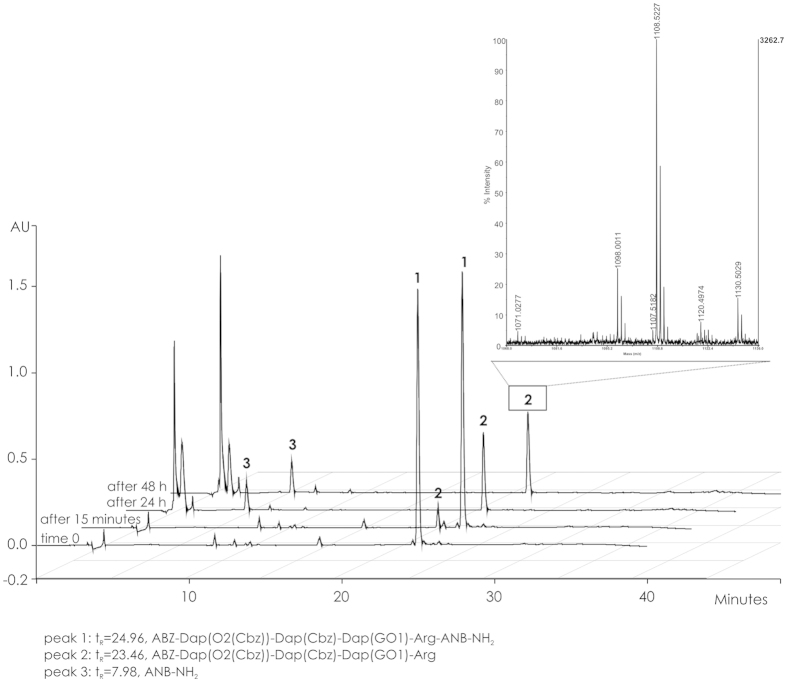
HPLC analysis of substrate 1 (1.97 μM) incubated with hNSP4 (14 nM) at different time points: 0, 15 min, 24 h and 48 h.

Incubation of substrate 1 (*t*_R_ = 24.96, MW 1271.34) with hNSP4 (Fig. 4) resulted in cleavage of the peptide bond located between Arg and ANB-NH_2_. The fluorescent fragment, ABZ-Dap(O2(Cbz))-Dap(Cbz)-Dap(GO1)-Arg-OH (*t*_R_ = 23.46, MW 1108.21), lacking its *C*-terminal quencher group, was no further degraded up to 48 h under the conditions of our assay (Fig. 4).

### Kinetic parameters

The obtained substrate 1 displayed high affinity (expressed as K_M_ reaching 7.6 × 10^−6 ^M) and moderate *k*_cat_ value of 1 s^−1^, and its specificity parameter value reached 1.3 × 10^5 ^M^−1^ × s^−1^ (Fig. S7).

### Selectivity

**Figure 5:**
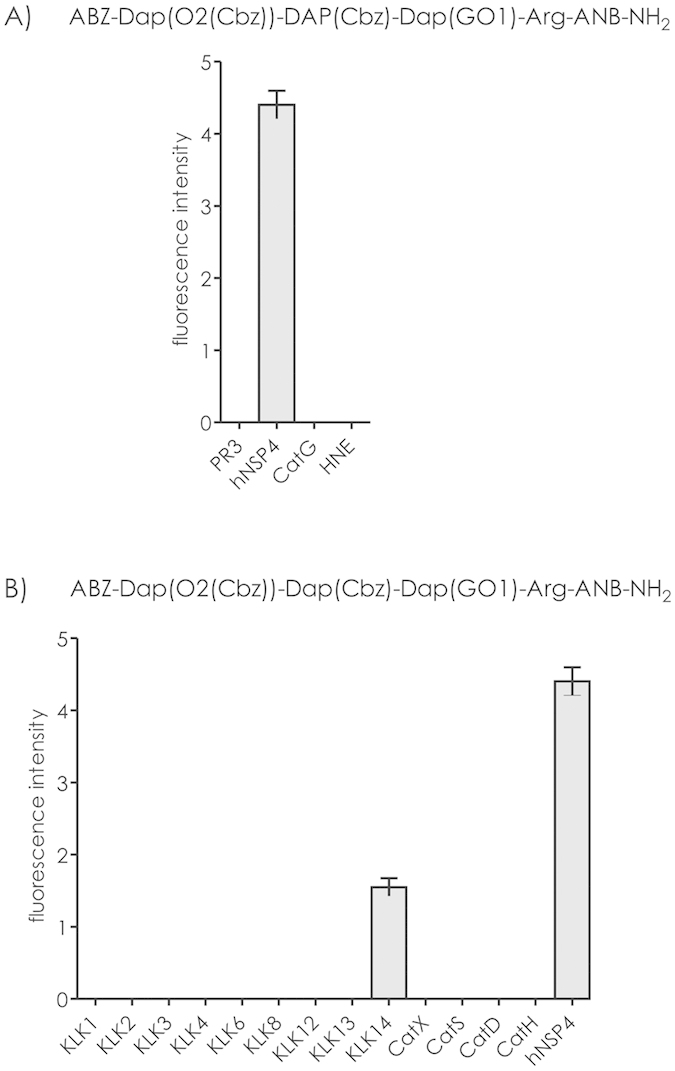
Incubation of substrate 1 (1.97 μM) with (**A**) neutrophil serine proteases (cathepsin G, elastase, and proteinase 3) at a concentration of 8.7 nM; (**B**) set of blood-associated proteases involving cathepsins and kallikreins.

The incubation of substrate 1 with neutrophil serine proteinases (CatG, HNE, or PR3) at a concentration of 10^−8 ^M produced no visible proteolysis (see Fig. 5A). Analogous experiment with set of proteases related to blood cells, including cathepsins and kallikreins, revealed that the developed substrate is susceptible to NSP4-mediated proteolysis and to a much lesser extend to degradation by KLK14. No other proteolytic enzyme at a concentration of 10^−8 ^M was able to cleave any of the peptide bonds of probe 1 at a noticeable level (Fig. 5B).

### Detection limit

The incubation of substrate 1 with decreasing amounts of hNSP4 yielded a visible fluorescence (signal-to-noise ratio of 3:1) at 10^−11 ^M of the studied enzyme (Fig. S8).

### Molecular model of substrate–enzyme interactions

**Figure 6:**
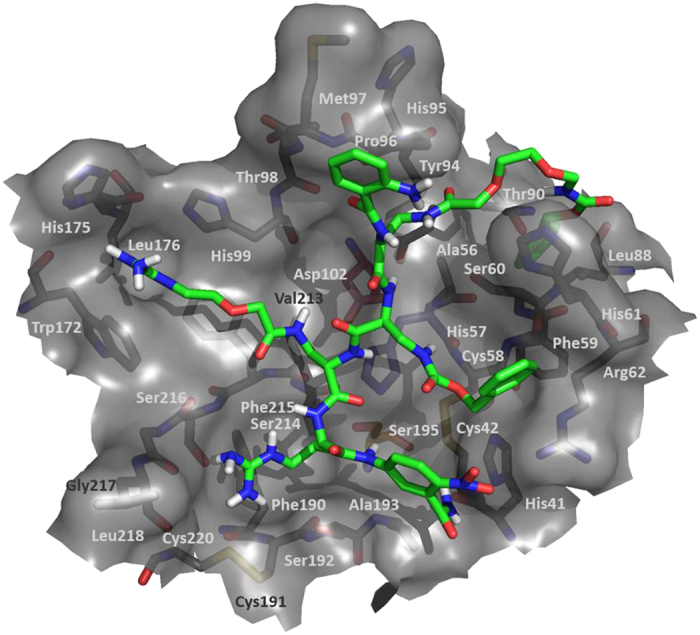
Proposed putative model of substrate 1 binding to the active site of NSP4 (based on 4Q7Z.pdb). The enzyme binding-site is presented as surface with the residues composing the binding pocket and catalytic triad (Ser195 – orange, His57 – blue, Asp102 - red) represented as sticks.

A molecular docking approach was employed to investigate the substrate 1-NSP4 binding mode. The most probable ligand-protein complex is presented in Fig. 6. The ANB residue is located near His41 and Arg62, and is able to create two types of interactions: π-π between the ANB and His41 aromatic rings and two hydrogen bonds between the amine group of ANB and the carbonyl oxygen of His41, and between nitro group of ANB and Arg62. The arginine residue in the X_1_ position is located in the Ser214 binding pocket and is stabilized by two hydrogen bonds with Ser216 and the backbone of Gly217. Additionally, it can form a cation – π interaction with the Phe190 residue. According to experimental data, the preferred residue in the X_2_ position is Dap(GO1). Our model shows that this residue is located in the cavity normally occupied by the substrate backbone. The guanidinium group of Dap(GO1) is located in the nest created by the side chains of His99, Trp172, His175 and Phe215 (S_4_ pocket), thus creating strong cation – π interactions. Additionally, we have found a hydrogen bond with the backbone of Trp172. The peptide bond between arginine (X_1_) and ANB-NH_2_ is located in the proximity of the Ser195 found within the catalytic triad in an orientation which allows for a nucleophilic attack at the carbonyl carbon. The most preferred residue in the X_3_ position is Dap(Cbz). It is located near the His57 residue of the catalytic triad and in this case we found backbone-backbone interactions. Additionally, we have found a cation – π interaction with Arg62. The most preferred residue in the X_4_ position is Dap(O2(Cbz)). The chain Dap(O2(Cbz)) is located in the vicinity of His61, however the phenyl ring of the carbobenzyloxy moiety is located in the nest created by Phe59, Leu88 and Thr90. We did not find any significant interactions of the ABZ residue with NSP4.

## Discussion

Proteases belong to one of the largest group of enzymes and have a broad range of activity and overlapping cleavage sequences/specificities. Chasing a single proteolytic enzyme in complex biological systems is a demanding challenge. In this work, the synthetic method used for the development of the library represents novel atypical peptidomimetics based on a submonomeric approach. The presence of a fluorogenic ABZ (2-amino benzoic acid) moiety and ANB-NH_2_ (amide of 5-amino-2-nitrobenzoic acid) fluorescence quencher pair facilitates in-solution fluorescence screening. The introduction of novel pseudo amino acid side chains such as those functionalized by different chemical entities of polyethylene glycol moieties covers a broad range of interactions between the enzyme studied and the selected substrate. The sequence revealed upon deconvolution of such DAPEG (Dap modified by PEG moiety) library supplement by typical amino acid residues is a novel approach, lacking any similarity to any work reported recently by our group. It has been constructed with two DAPEG units with different functional group on the side chains. Such diversified functional groups together with their glycol side chains offer multiple contacts between the enzyme and the selected substrate. As indicated, in position X_4_ a long polyethylene glycol side chain terminated by an aromatic group is preferred over other chemical entities at this position. In general, this result is in agreement with data provided by Perera *et al*.^15^ since NSP4 prefers an aromatic amino acid in this position. However, the side chain length is an important factor that makes a significant difference between Phe/Tyr and Dap(O2(Cbz)). In the light of data summarized in Table 1 that provides published up to date NSP4 substrate sequences, position P4 is dominated by hydrophobic amino acid residues (hCha, Phe).

Positions X_3_ and X_2_ are also occupied by atypical residues; however, the short length of Dap(Cbz) in the X_3_ position could be considered an aromatic amino acid imitation since Dap(GO1) in X_2_ structurally resembles homologous elongated form of Arg. As presented in Table 1, combinations of amino acid residues with hydrophobic and basic side chains (Ile/Arg, Phe(4-guanidine)/Oic, Lys/Pro) are preferred in the P2/P3 positions. A similar pattern is observed in our newly developed substrate where Dap(Cbz) in position P3 is followed by pegylated analog of Arg (Dap(GO1) in position P2.

The kinetic parameters, especially k_cat_/K_M_ ratio (over 1.31 × 10^5 ^M^−1^ × s^−1^) of the developed compound are superior over other NSP4 substrates reported thus far (summarized in Table 2) and display specificity 10-fold greater than a substrate described by Lin *et al*.^18^ and 4-times higher than a substrate selected by Kasperkiewicz *et al*.^19^

The presence of factionalized glycol side chains in the Dap scaffold of these peptidomimetics offers high selectivity over other proteolytic enzymes. This was demonstrated using a panel of over 20 neutrophil-related proteinases which included NSPs and a family of human kallikreins recently found to be expressed in neutrophils (Fig. 5)^20^. The kinetic parameters of substrate 1 and together with its low (subnanomolar) detection limit make this peptidomimetic an excellent tool to study NSP4 activity in biological material. Until now, there is a limited set of data regarding the NSP4 specificity. According to Perera *et al*., the non-primed segment (spanning residues P4 - P1) of the peptide chain interacting with NSP4 should be composed as follows: Gly-Ile-Pro-Arg. The substrate sequence obtained in this study did not display any obvious similarity, except for the Arg residue in the P1 position, which ensures the proper substrate recognition by the enzyme. The exceptional proteolytic stability of the cleaved *N*-terminal fragment of the peptidomimetic chain is worth highlighting, as this clearly indicates that the only peptide bond susceptible to NSP4-mediated hydrolysis is located between the Arg and amide of ANB. In the molecular docking model, the binding of the P1 arginine side chain is stabilized by the interactions of the guanidinium group with residues building the external surface of S1 pocket, and the NH_2_ of ANB group forming a hydrogen bond with His41.

The low solubility of small peptidomimetics or modified peptides often hampers their use in well-designed experiments. This is not the case for this class of substituted Dap derivatives as exemplified by our compound 1 which displays excellent solubility in water.

The unnatural character and considerable size of the side chains of the presented NSP4 substrate (compound 1) together with its significant differences from previously reported NSP4 substrate specificity in the P4-P2 pockets (Gly-Ile-Pro) provoked us to investigate alternative modes of binding. According to Perera *et al*.^16^ arginine and serine residues located at the *C*-terminal part of the ligand display the highest activity toward NSP4. This result is consisted with the proposed model of binding. The arginine residue in the P1 position is long enough to create a hydrogen bond with Ser216 and the backbone of Gly217. A small residue such as ANB is able to create more hydrogen bonds (with His41 and Arg62) in comparison to serine in discussed position (P1′). Our results indicate that the most preferred residue in the P2 position is Dap(GO1) while Perera *et al*.^16^ suggest arginine residue in this position. With the proposed mode of binding Dap(GO1) is located in the cavity normally occupied by the substrate backbone. This cavity is highly hydrophobic, created by the following residues: His99, Trp172, His175 and Phe215 (S4 pocket). None of the investigated residues fits better to the described cavity than Dap(GO1). Dap(MO1) and Dap(MO2) can create a set of hydrophobic interactions, however those residues are not able to create any hydrogen bonds. Dap(O1), Dap(O2), Dap(HO1) and Dap(HO1) are able to create hydrogen bonds, however, due to their high conformational flexibility, they are not able to maintain stable and strong interactions. The side chain of the residue in the P2 position cannot be too long, since we did not observe significant activity with Dap(GO2). According to the proposed model of binding, the most preferred residue in the P3 position should be Dap(GO1) and Dap(GO2) since both residues are able to form a salt bridge with Arg62. However, our experimental results showed the opposite effect indicating that Dap(Cbz) was the most preferred residue at the P3 position. The residue at the P3 position is located adjacent to the catalytic triad (especially H57). It might be suspected that the role of the P3 residue is also to protect the catalytic triad from external influence. The most interesting result was obtained in regards to the P4 position which seems to be occupied by an aromatic residue. Moreover, the chain length seems to be important in the activation process: the short chain (Dap(Cbz)) would interact with His61 and/or Pro96 while its elongation would weaken this interaction as a slight decrease in the NSP4 activity with Dap(O1(Cbz)) was observed. When the side chain was long enough to reach the protein surface we observed a major increase in NSP4 activity. Hence, the proposed binding place of the P4 residue is located beyond the ligand binding site; thus position P4 could be considered as a target for the design of specific and highly selective ligands for the NSP4.

To our best knowledge, the presented approach is unique and could effectively be applied for the development of novel selective synthetic substrates of any proteolytic enzyme. Our preliminary data indicate that following this approach novel substrates with superior kinetic parameters could be obtained for other proteolytic enzymes with canonical substrate binding pockets such as trypsin or matriptase (unpublished data). Iterative screening of DAPEG librarys could be applied to any proteolytic enzyme with known primary specificity. Fixing the P1 residue assures primary specificity while introducing variability of chemical entities that differ by length, charge and hydrophobicity at other positions to yield the optimal substrate sequence.

The method proposed here enriches the panel of previously known methods used to investigate secondary binding sites (exosites) on the surface of proteases. So far, only the exosite cellular libraries of peptide substrates (eCLiPS) approach proposed by the Daugherty group deals with protease exosite identification^21^. In this work the cellular libraries of peptide substrates (CLiPS) method was modified to support the screening process of peptides that enhance the proteolysis rate of a model peptide substrate. Both the peptide substrate and a peptide library of exosite ligands were expressed on cell surface of *E. coli.* The eCLiPS approach characterizes the functions of protease exosites and recognizes peptide sequences that are crucial for boosting protease specificity for natural protein substrates. This is not the issue in the method developed by us since it instead yields the secondary specificity of a protease forming a contact with distant subsites.

This novel class of peptidomimetic offers practically unlimited possibilities for generation of new peptide-like molecules with diverse application not only limited to protease research. An enormous number of protecting groups that could be attached to functionalized polyethylene glycol moieties in combination with different glycol length creates an excellent chemical repertoire for the development of new kinds of molecules with diverse activities.

## Materials and Methods

The synthesis of the ANB-based library or peptides was initiated by the deprotection of the amino groups of the resin with 20% piperidine in DMF and the coupling of 5-amino-2-nitrobenzoic acid using a mixture of N,N,N′,N′-tetramethyl-O-(benzotriazol-1-yl)uronium tetrafluoroborate (TBTU)/4-dimethylaminopyridine (DMAP). The resin was washed twice in N methylmorpholine. Next, 2 equiv. of ANB was dissolved in DMF and 2 equiv. of TBTU was added, followed by 1 equiv. of DMAP. The obtained solution was added to the resin, and after 30 s, 4 equiv. of N,N-diisopropylethylamine (DIPEA) was added. The whole mixture was stirred for 3 h. The solution was filtered off, and the resin was washed with DMF. The procedure was repeated three times. Next, the first amino acid residue was coupled with a method described in ref. 22. A ninefold excess was applied to the active resin sites as follows: the amino acid was dissolved in pyridine (10 ml pyridine to 1 g peptidyl-resin). The whole solution was mixed until the temperature of −15 °C was reached and then 9 equiv. of POCl_3_ was added. The mixture was successively stirred for 20 min at −15 °C, 20 min at room temperature, and 6 h in an oil bath. After deprotection with 20% piperidine in DMF, the peptide chain was elongated as follows: resin was divided into 18 equal parts, and in six positions, the amino acid derivatives such as Fmoc-Asp(tBu), Fmoc-Ser(OtBu), Fmoc-Ser(OMe), Fmoc-Dap(Cbz), Fmoc-Agp(Boc)_2_, Fmoc-Dap(Boc) were introduced—where Dap is (S) 2,3-diaminopropionic acid and Agp ((S)2-amino-3-guanidino-propionic acid)—using a standard Fmoc synthesis protocol. To each of remaining 12 portions, Fmoc-Dap(Mtt) was introduced and 4-methyltrityl (Mtt) was removed using procedure described in ref. 23 (2% TFA in DCM with addition of 1% triisopropylsilane). Such mixture was added to every 12 portions and stirred for 15 min. Completeness of Mtt removal was verified by adding pure TFA and yellow color development was monitored to indicate the presence of free Mtt groups. The whole procedure was repeated until no absorbency increase monitored at 410 nm was recorded. Next, DIPEA/DMF solution was added to each system for 15 min, resulting in 12 portions of Fmoc-Dap-Arg-ANB-polymer. To each of the above resin aliquots, the monoprotected heterobifunctional polyethylene glycol moieties (PEG) were coupled using equimolar amounts of PEG/DIPCI/HOBt in DMF/NMP (1:1, v/v) solution. The following protected PEG derivatives were used: 5-(t-butyloxycarbonyl-amino)-3-oxapentanoic acid (further abbreviated as O1), 8-(t-butyloxycarbonyl-amino)-3, 6-dioxaoctanoic acid (O2), 5-[N-t-butyloxycarbonyl-N′-(2,2,4,6,7-pentamethyldihydro benzofuran-5-sulfonyl)]amidino-3-oxapentanoic acid (GO1), 8-[N-t-butyloxycarbonyl-N′-(2,2,4,6,7-pentamethyldihydrobenzofuran-5-sulfonyl)]amidino-3,6-dioxaoctanoic acid (GO2), 5-(benzyloxycarbonyl-amino)-3-oxa-pentanoic acid (O1(Cbz)), 8-(benzyloxycarbonyl-amino)-3,6-dioxaoctanoic acid dicyclohexylamine (O2(Cbz)), 2-(2-tert-butoxyethoxy) acetic acid (HO1), 2-(2-(2-tert-butoxyethoxy)ethoxy) acetic acid (HO2), 3,6-dioxaoctanedioic acid 1-tert-butyl ester (C01), 3,6,9-Trioxaundecandioic acid 1-tert-butyl ester (CO2), 5-methoxy-3-oxapentanoic acid (MO1), 8-methoxy-3,6-dioxaoctanoic acid (MO2). Completeness of the coupling was monitored using a Kaiser test. Lack of free amino groups allowed us to move to the next step of library synthesis in which all resin portions were mixed and again divided into 18 parts. The whole procedure was repeated twice. Finally, the tert-butyloxycarbonyl derivative of 2-amino benzoic acid (Boc-ABZ-OH) was attached to the N-terminal amino group using the standard coupling method described above.

After completing the synthesis, the sublibraries or individual peptidomimetics were cleaved from the resin, using a TFA/phenol/triisopropylsilane/H2O mixture (88:5:2:5, v/v)^24^. The purity of the synthesized compounds and the correctness of the synthesis were confirmed using an RP-HPLC ChromNAV (Jasco, Japan) equipped with a Kromasil 100 C8 column (Knauer, Germany) equipped with a UV-Vis detector and a fluorescence detector. A linear gradient from 10% to 90% B within 45 min was applied (A: 0.1% TFA; B: 80% acetonitrile in A). The peptides were monitored at 226 nm. The molecular weights of the synthesized peptides and peptide libraries were confirmed by analysis of the mass spectra which were recorded on a Biflex III MALDI TOF mass spectrometer (Bruker Daltonics, Germany) using α-cyano-4-hydroxycinnamic acid as a matrix.

### NMR analysis

All NMR spectra were recorded in H_2_O/D_2_O (9:1 v:v) at 298 K using the Bruker AVANCE III 700 MHz (Bruker Daltonics, Bremen, Germany). The concentration of the sample was 2.12 × 10^−4 ^M (0.7 ml). For the studied compound, the following spectra were recorded: 1D proton spectrum and 2D TOCSY and ROESY at mixing times of 80 ms and 200 ms as well as ^1^H-^13^C HSQC.

### Preparation of the peptide libraries

The peptide libraries were synthesized using a portioning-mixing method. Initially, 17.7 g of the solid support (TentaGel S RAM) was used for the first library, i.e. ABZ-X4-X3-X2-X1-ANB-NH_2_. A twofold molar excess of amino acid or appropriate monoprotected PEG derivative was used for the coupling. The other synthetic methods used were as described above.

### Enzymatic studies

For the enzymatic studies, we used human NSP4 expressed in a Pichia pastoris expression system as described in ref. 15. The NSP4 concentration used in the deconvolution of the library was 1.46 × 10^−9 ^M. The bovine β-trypsin (Sigma Chem. Co., USA) concentration was determined by spectrophotometric titration with 4-nitrophenyl-4′-guanidinobenzoate (NPGB) at an enzyme concentration oscillating around 10^−6 ^M. Standardized trypsin solution was used to titrate BPTI (Sigma Chem. Co., USA) which in turn served to determine the solution concentrations of the produced NSP4. The substrate ABZ-Met-Phe-Pro-Arg-ANB-NH_2_ recently developed in our lab was used. The deconvolution of peptide libraries was carried out by the iterative solution method^25^. The lyophilizates obtained for each sublibrary were dissolved in dimethyl sulfoxide (DMSO) to a final concentration of 5 mg/ml, and then diluted tenfold in an assay buffer. The fluorescence tests were performed using a FluoroStar OMEGA fluorescent microplate reader (BMG, Germany). The excitation and emission wavelengths were 320 nm and 410 nm for the ABZ/ANB. Enzymatic hydrolysis of the peptide was performed in 20 mM Tris-Cl buffer supplemented by 150 mM NaCl (pH 7.4) at 37 °C and continued over 30 min.

All other enzymes (purchased from RD Systems, USA) were tested in concentration 2 × 10^−9 ^M in 20 mM Tris-Cl buffer supplemented by 150 mM NaCl (pH 7.4) at 37 °C for 30 min. Same conditions as above were applied to the assay.

### Determination of kinetic parameters

Assay conditions for the determination of the Michaelis constants (K_M_) and catalytic constants (k_cat_) were as noted above. The specificity constants (k_cat_/K_M_) were calculated from k_cat_ and K_M_ values. Measurements were performed with an enzyme concentration of 1.46 × 10^−9 ^M. Three to five measurements were carried out for each compound (systematic error expressed as a standard deviation never exceeded 20%). The calculated initial hydrolysis rates were used as a measure of the substrate activity of the investigated peptides. All details of kinetic studies and the method of calculating kinetic parameters have been described elsewhere^26^.

### Determination of proteolytic cleavage patterns

For each sample of diluted substrate (concentrations 1.97 × 10^−6 ^M), the appropriate amount of enzyme (1.46 × 10^−8 ^M) was added, and the solution was incubated for 5, 30, and 60 min. The progress of the proteolytic reaction was monitored by RP-HPLC. The individual peaks resulting from the hydrolysis reaction were collected and identified using the mass spectra which were recorded on a Biflex III MALDI TOF mass spectrometer (Bruker Daltonics, Germany) using α-cyano-4-hydroxycinnamic acid as a matrix.

### Molecular docking

The in silico study was performed using AutoDock Vina 1.1.2 molecular docking software^27^. For the optimization of compound 1 model geometry the MM2 force field was employed. The simulation of protein-substrate interaction was based on the crystal structure of NSP4 with inhibitor (Phe-Phe-Arg-chloromethylketone; 4Q7Z.pdb)^18^. Initially the inhibitor and water coordinates were removed from the protein structure, and AutoDockTools 1.5.6 (Scripps Research Institute, USA) was employed to prepare the receptor and ligand models for docking studies. The molecular docking of flexible ligand to the rigid protein active center defined by the 50 × 50 × 20 Å grid box with a center at −14.5, −5.0, −1.7 was performed with the default settings.

## Additional Information

**How to cite this article**: Wysocka, M. *et al*. PEGylated substrates of NSP4 protease: A tool to study protease specificity. *Sci. Rep.* **6**, 22856; doi: 10.1038/srep22856 (2016).

## Supplementary Material

Supplementary Information

## Figures and Tables

**Table 1:** Substrates sequence alignment according to Schechter Berger notation.

No	P5	P4	P3	P2	P1	P1′	P2′	P3′	P4′
1	MCA-	PEG	Ile	Arg	Arg	Ser	Ser	Tyr	Ser
2	Ac	hCha	Phe(4-guanidine)	Oic	Arg	ACC	–	–	–
3	ABZ	Dap(O2(Cbz)	Dap(Cbz)	Dap(GO1)	Arg	ANB-NH_2_	–	–	–
4	MCA-Gly	Phe	Lys	Pro	Arg	Ser	Arg	Pro	Lys(Dnp)-arg-arg

Where Oic is octahydroindole-2-carboxylic acid, Phe(4-guanidine) 4-gaunidyl-l- phenylalanine, ACC 7-amino-4-carbamoylmethylcoumarine, Dnp 2,4-dinitrophenyl, MCA (7-methoxycoumarin-4-yl)acetyl acid, hCha cyclohexylo-l-homoalanine, arg = D-arg and PEG -8- amino)-3,6-dioxaoctanoic acid.

**Table 2:** Kinetic parameters of reported artificial substrates of NSP4.

No	Sequence	k_cat_ [s^−1^]	K_M_ [μM]	k_cat_/K_M_ M^−1^ × s^−1^	Ref.
1	MCA-PEG-Ile-Arg-**Arg-Ser**-Ser-Tyr-Ser-Phe-Lys(Dnp)-Lys	0.136 ± 0.008	13.8 ± 3.3	10,000	18
2	Ac-hCha-Phe(4-guanidine)-Oic-Arg-ACC	2.0 ± 0.5	61.0 ± 9.1	32,786	19
3	ABZ-Dap(O2(Cbz))-Dap(Cbz)-Dap(GO1)-Arg-ANB-NH_2_	1.0 ± 0.1	7.6 ± 0.5	131,579	
4	MCA-Gly-Phe-Lys-Pro-Arg-Ser-Arg-Pro-Lys(Dnp)-arg-arg	ND	ND	ND	16

Where Oic is octahydroindole-2-carboxylic acid, Phe(4-guanidine) 4-gaunidyl-l- phenylalanine, ACC 7-amino-4-carbamoylmethylcoumarine, Dnp 2,4-dinitrophenyl, MCA (7-methoxycoumarin-4-yl)acetyl acid, hCha cyclohexylo-l-homoalanine, arg = D-arg and PEG -8- amino)-3,6-dioxaoctanoic acid.
