# Supplementary material for: PEGylated substrates of NSP4 protease: A tool to study protease specificity
Source: Sci Rep. 2016 Mar 9;6:22856. doi: 10.1038/srep22856 (PMC4783772; doi:10.1038/srep22856)
Supplement: Supplementary Information [file srep22856-s1.pdf]

Suplement materials for manuscritpt PEGylated substrates of NSP4 protease: A tool to study protease specificity.

Wysocka Magdalena, Gruba Natalia, Grzywa Renata, Artur Giełdoń, Bąchor Remigiusz, Brzozowski Krzysztof, Sieńczyk Marcin, Jenne Dieter, Szewczuk Zbigniew, Rolka Krzysztof, Lesner Adam

Mass spectrometry

All ESI-MS experiments were performed on a micrOTOF-Q mass spectrometer (Bruker Daltonics, Bremen, Germany) equipped with standard ESI source. The instrument was operated in the positive-ion mode and calibrated with the Tunemix™ mixture (Agilent Technologies, Palo Alto, CA, USA). The scan range was between 200 – 1700 m/z. The mass accuracy was better than 5 ppm. Samples were dissolved in 200 µL of the water/MeCN/HCOOH (50:50:0.1, v/v/v) mixture. Analyte solution was infused at a flow rate of 3 µl/min. The instrument parameters were as follows: scan range: 50-1600 *m/z*; drying gas: nitrogen; flow rate: 4.0 L/min, temperature: 200 ºC; potential between the spray needle and the orifice: 4.2 kV.

CID

The doubly charged precursor ion was selected on the quadrupole and subsequently fragmented in the hexapole collision cell. Argon was used as a collision gas. The obtained fragments were registered as an MS/MS (tandem mass spectrometry) spectrum. The collision voltage (10-26 V) was optimized for the best fragmentation. For MS spectra analysis, a Bruker Compass DataAnalysis 4.0 software was used.

Suplement materials for manuscritpt Pegylated substrates of NSP4 protease – new tool for study proteases exosites.

Wysocka Magdalena, Gruba Natalia, Grzywa Renata, Bąchor Remigiusz, Brzozowski Krzysztof, Sieńczyk Marcin, Jenne Dieter, Szewczuk Zbigniew, Rolka Krzysztof, Lesner Adam

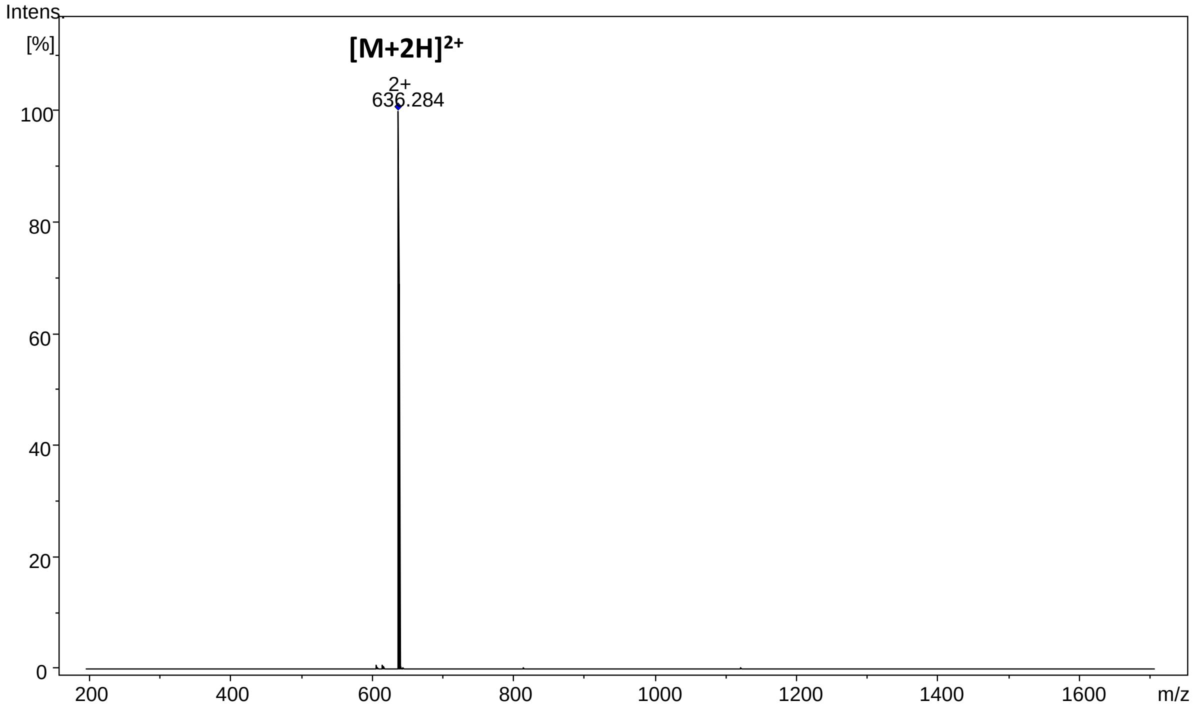

Fig S1. ESI MS spectra of analyzed compound. Paternal ion 636,285 collision energy 10eV.

Wysocka Magdalena, Gruba Natalia, Grzywa Renata, Bąchor Remigiusz, Brzozowski Krzysztof, Sieńczyk Marcin, Jenne Dieter, Szewczuk Zbigniew, Rolka Krzysztof, Lesner Adam

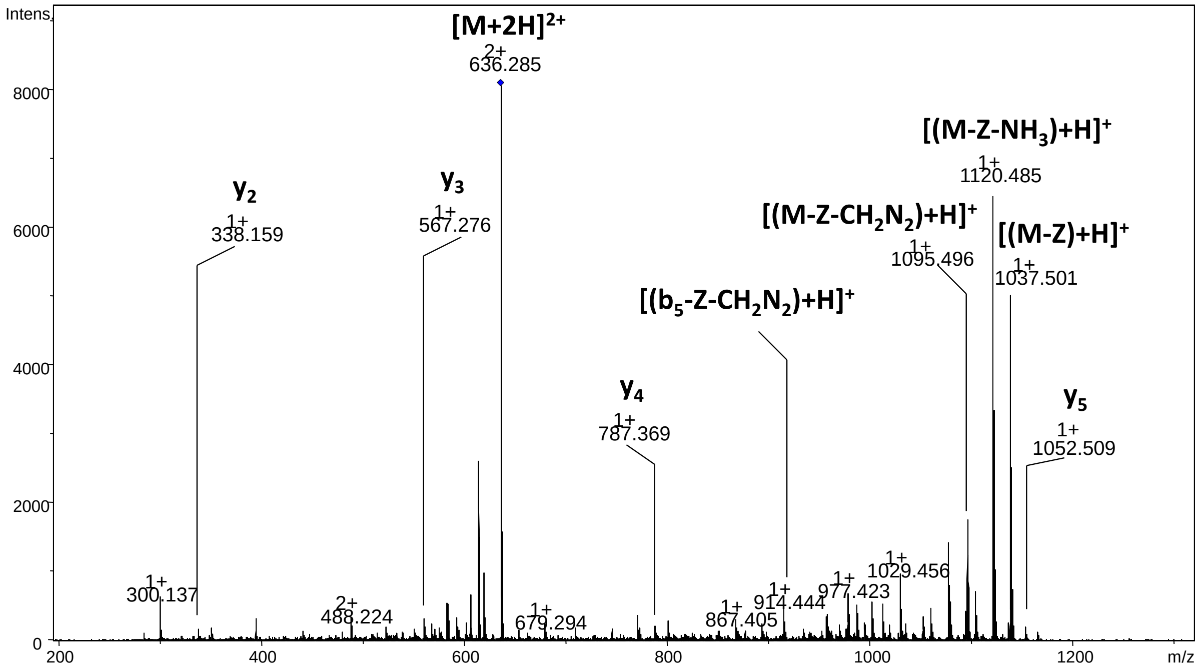

Fig S2. ESI-MS/MS spectra of analyzed compound. Paternal ion 636,285 collision energy 26 eV.

Wysocka Magdalena, Gruba Natalia, Grzywa Renata, Bąchor Remigiusz, Brzozowski Krzysztof, Sieńczyk Marcin, Jenne Dieter, Szewczuk Zbigniew, Rolka Krzysztof, Lesner Adam

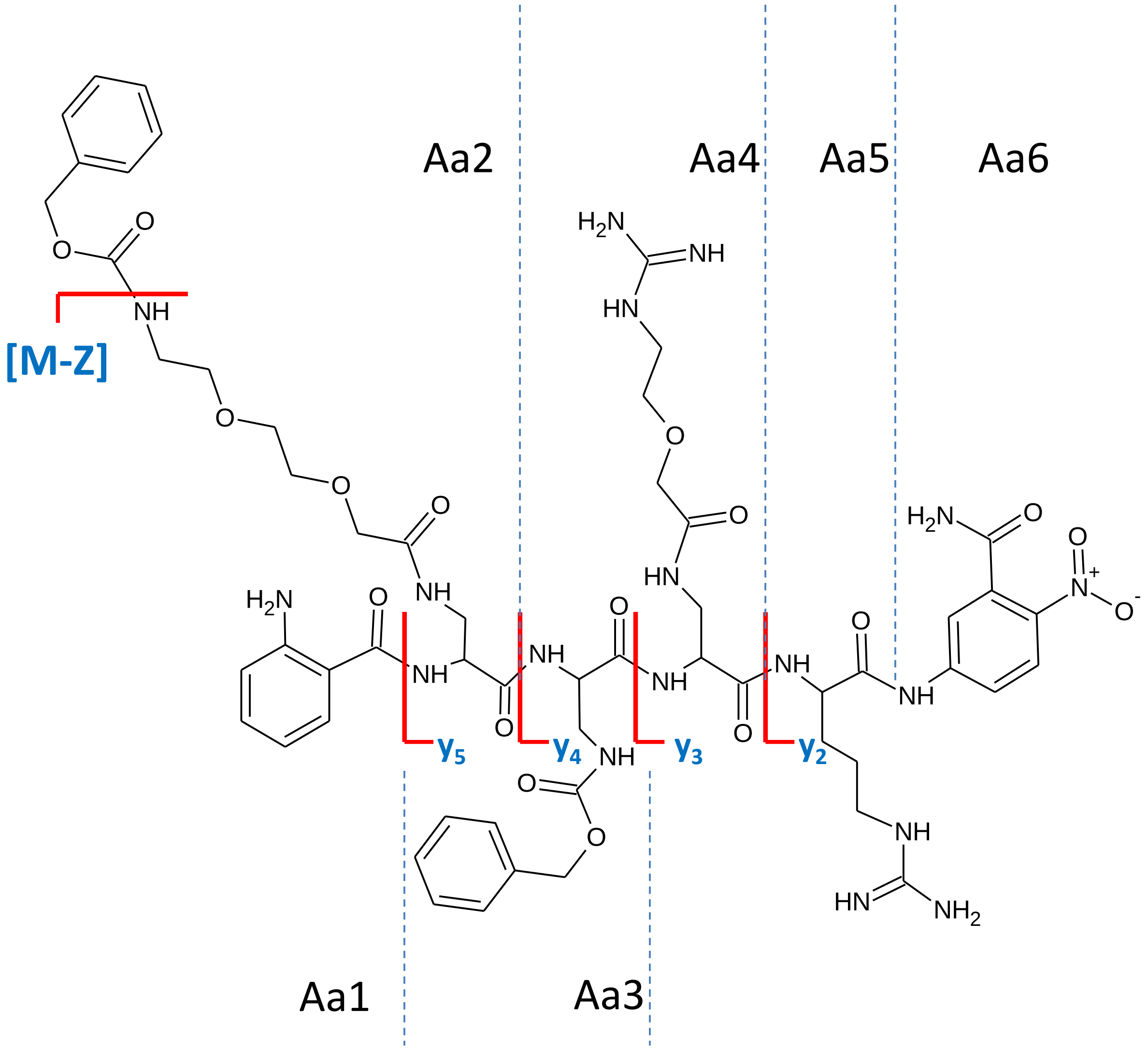

Fig S3. Schematic presentation of compound 1 fragmentation pattern.

NMR studies

All NMR spectra were recorded in H_2O/D_2O (9:1 v:v) or DMSO at 298 K. The concentration of the sample was 2.12 $\times 10^{-4}$ M.(0.7 ml). For the peptide studied the following spectra were recorded 1D proton spectrum and 2D TOCSY and ROESY at mixing times of 80 and 200 ms and additionally ^{1}H-^{13}C HSQC.

Attempts of resolving the molecule sequence (ABZ^{6}-Dap(O2(Cbz))^{5}-Dap(Cbz)^{4}-Dap(GO1)^{3}-Arg^{2}-ANB-NH_2^{1}) were made using 2D H^1 NMR. The first was recognized the characteristic spin system of Arg (residue 2). Next on the basis of sequential NOE effects two substituted Dap residues linked via peptide bond were assigned (residues 3, 4 and 5 respectively). The chemical shifts of side chain HN protons of Dap (diaminopropionic acid residues) were determined by couplings with HN deriving from the backbone. The chemical shift of HN of ANB-NH_2 (residue 1) was established on the basis of the coupling with HN of Arg. Protons of aromatic ring of ANB-NH_2 were assigned by the coupling with HN of the same moiety. Residue 6 presence were visible as aromatic protons coupled with amide protons of Dap residue.

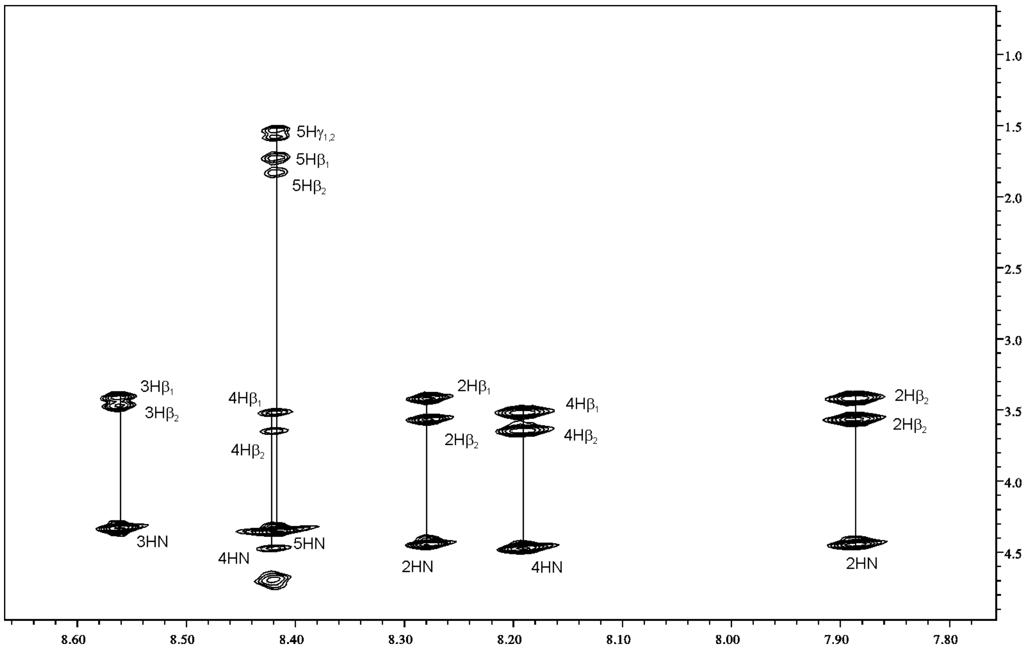

Fig S4. Diagnostic region of TOCSY spectrum with marked spin systems of aminoacid residues present in the analyzed molecule

Suplement materials for manuscritpt Pegylated substrates of NSP4 protease – new tool for study proteases exosites.

Wysocka Magdalena, Gruba Natalia, Grzywa Renata, Bąchor Remigiusz, Brzozowski Krzysztof, Sieńczyk Marcin, Jenne Dieter, Szewczuk Zbigniew, Rolka Krzysztof, Lesner Adam

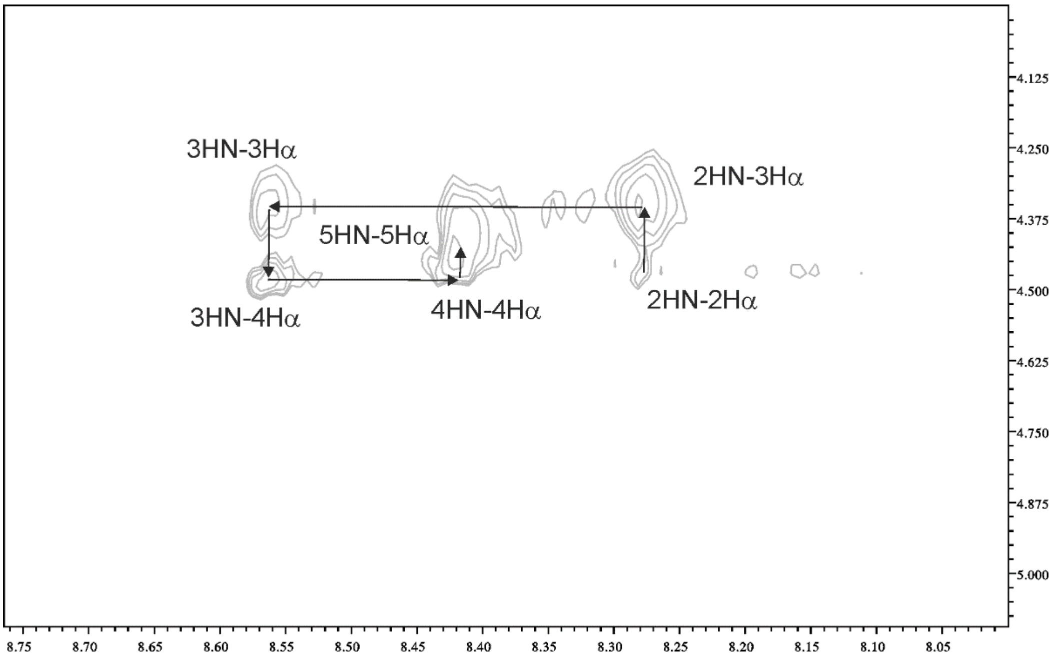

Fig S5 Diagnostic region of ROESY spectrum with marked sequential couplings

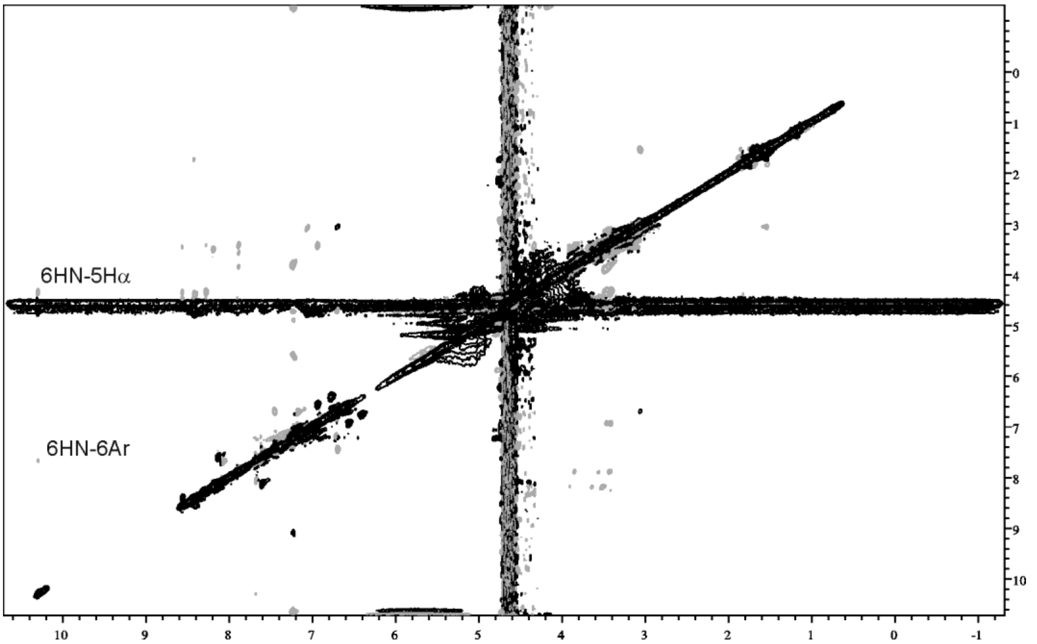

Fig S6. ROESY spectrum with marked coupling of ABZ.

Kinetics

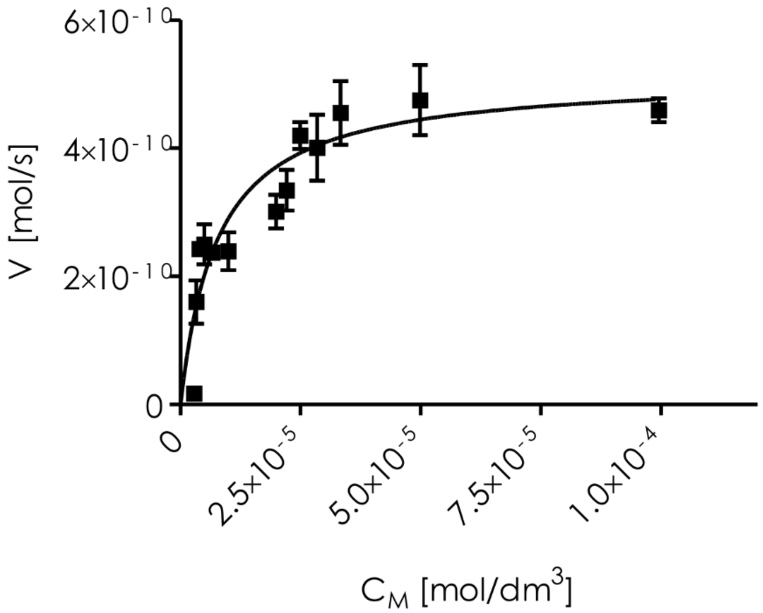

Fig S7. Michealis menten curve obtained for compound 1.

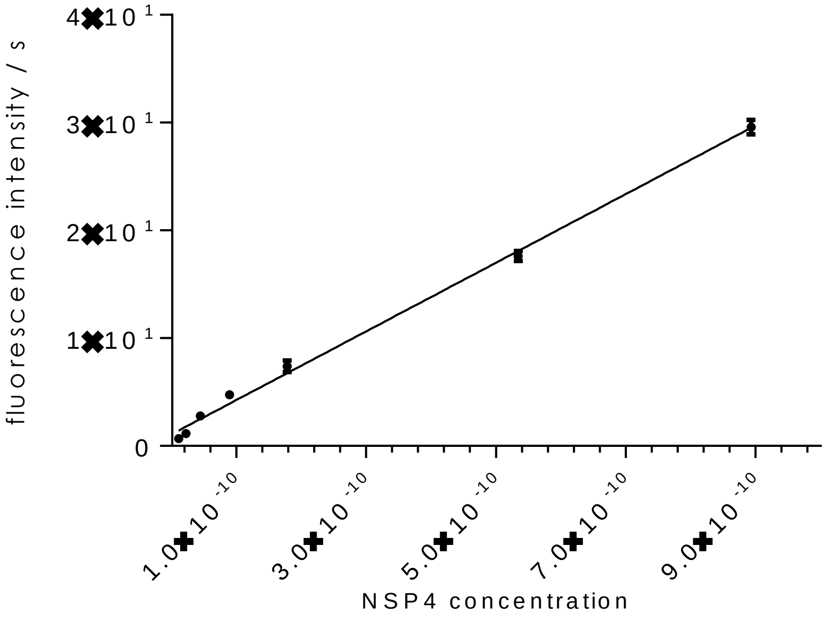

Fig S8 Titration of increasing hNSP4 with constant amount (1.97 of substrate 1.

Suplement materials for manuscritpt Pegylated substrates of NSP4 protease – new tool for study proteases exosites.

Wysocka Magdalena, Gruba Natalia, Grzywa Renata, Bąchor Remigiusz, Brzozowski Krzysztof, Sieńczyk Marcin, Jenne Dieter, Szewczuk Zbigniew, Rolka Krzysztof, Lesner Adam

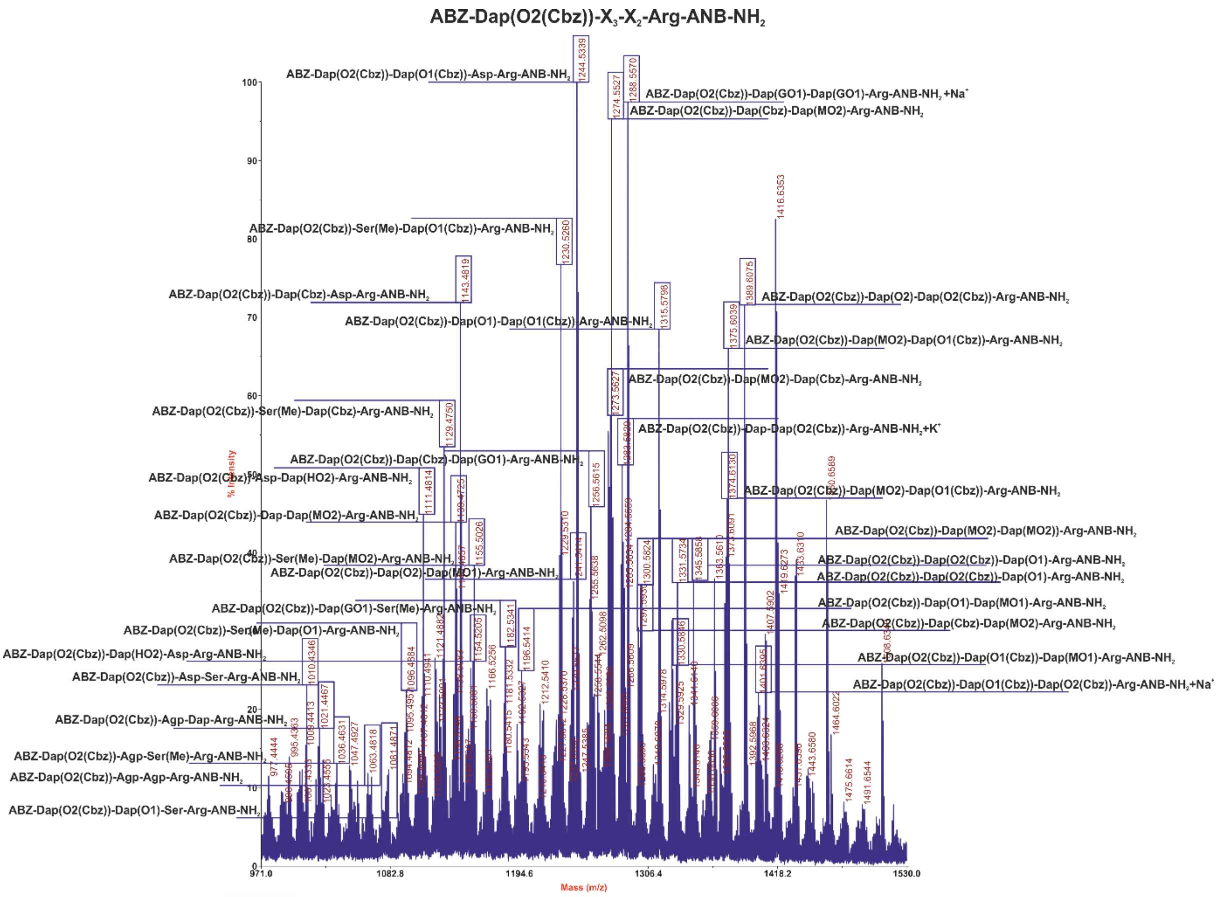

Fig S9. HR MS MALDI analysis of library with general formula ABZ-Dap(O2(CBZ)-X3-X2-Arg-ANB-NH2.

Suplement materials for manuscritpt Pegylated substrates of NSP4 protease – new tool for study proteases exosites.

Wysocka Magdalena, Gruba Natalia, Grzywa Renata, Bąchor Remigiusz, Brzozowski Krzysztof, Sieńczyk Marcin, Jenne Dieter, Szewczuk Zbigniew, Rolka Krzysztof, Lesner Adam

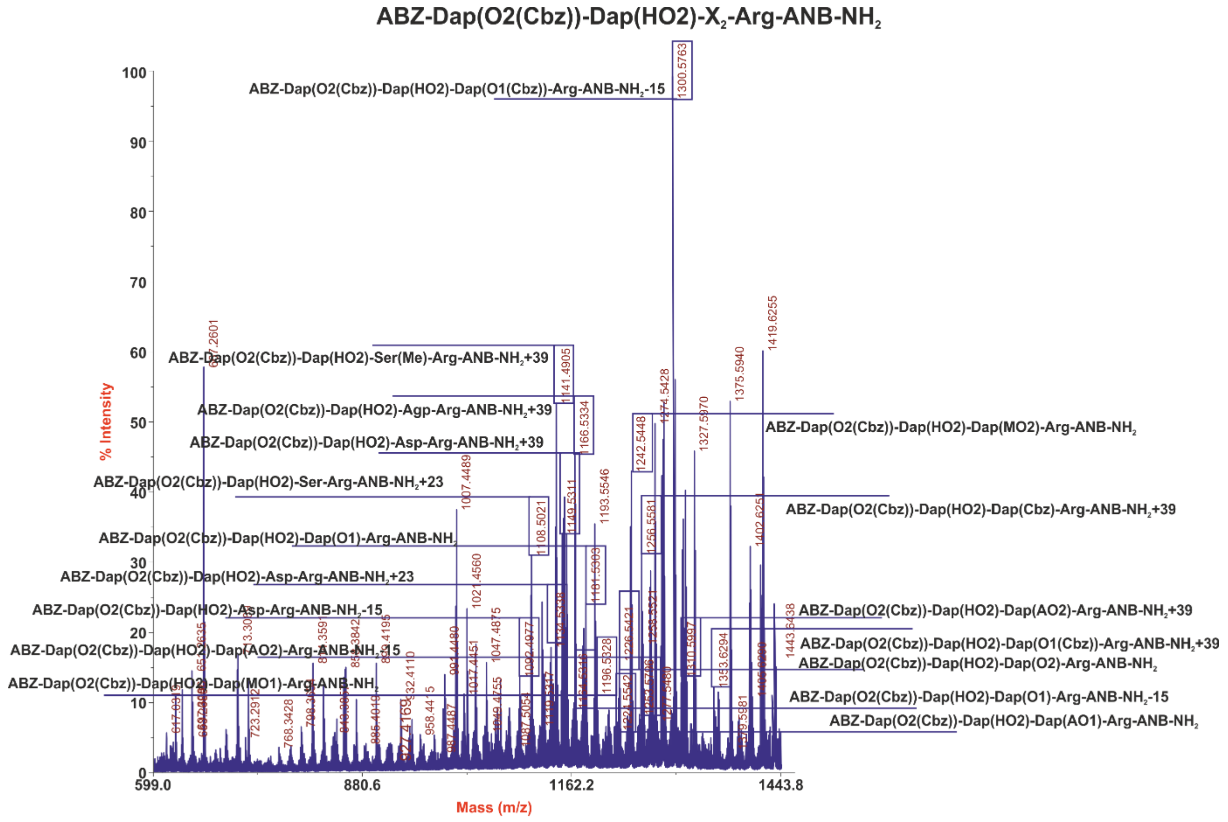

Fig S10. HR MS MALDI analysis of library with general formula ABZ-Dap(O2(CBZ))-Dap(HO2)-X2-Arg-ANB-NH_2.

Suplement materials for manuscritpt Pegylated substrates of NSP4 protease – new tool for study proteases exosites.

Wysocka Magdalena, Gruba Natalia, Grzywa Renata, Bąchor Remigiusz, Brzozowski Krzysztof, Sieńczyk Marcin, Jenne Dieter, Szewczuk Zbigniew, Rolka Krzysztof, Lesner Adam

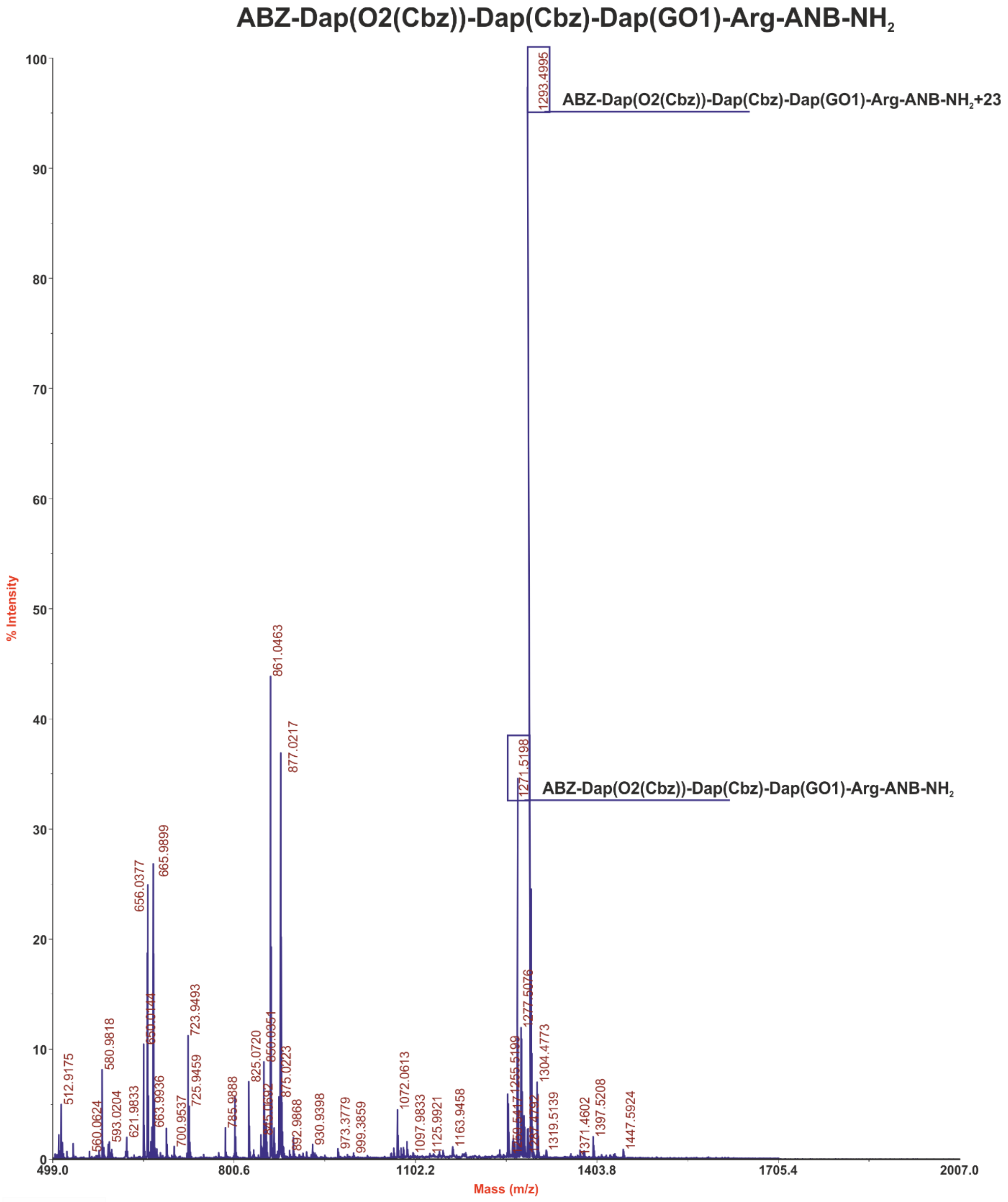

Figure S11. HR MS MALDI analysis of library with general formula ABZ-Dap(O2(CBZ)-Dap(Cbz)Dap(GO1)-Arg-ANB-NH_2.
